# Machine learning enhanced cell tracking

**DOI:** 10.3389/fbinf.2023.1228989

**Published:** 2023-07-14

**Authors:** Christopher J. Soelistyo, Kristina Ulicna, Alan R. Lowe

**Affiliations:** ^1^ Department of Structural and Molecular Biology, University College London, London, United Kingdom; ^2^ Institute for the Physics of Living Systems, London, United Kingdom; ^3^ Alan Turing Institute, London, United Kingdom

**Keywords:** machine learning (ML), computer vision, tracking, cell tracking, bioimage analysis, optimisation

## Abstract

Quantifying cell biology in space and time requires computational methods to detect cells, measure their properties, and assemble these into meaningful trajectories. In this aspect, machine learning (ML) is having a transformational effect on bioimage analysis, now enabling robust cell detection in multidimensional image data. However, the task of cell tracking, or constructing accurate multi-generational lineages from imaging data, remains an open challenge. Most cell tracking algorithms are largely based on our prior knowledge of cell behaviors, and as such, are difficult to generalize to new and unseen cell types or datasets. Here, we propose that ML provides the framework to learn aspects of cell behavior using cell tracking as the task to be learned. We suggest that advances in representation learning, cell tracking datasets, metrics, and methods for constructing and evaluating tracking solutions can all form part of an end-to-end ML-enhanced pipeline. These developments will lead the way to new computational methods that can be used to understand complex, time-evolving biological systems.

## 1 Introduction

Understanding how cells self-organize to become tissues and whole organisms is one of the most fundamental questions of biology. Indeed, single cell biology has the potential to illuminate processes from development and regeneration to diseases such as cancer. A predicate of quantifying cell biology in space and time is a suite of computational tools that can extract measurements from the myriad sources of experimental data. These include algorithms to detect cells, measure properties such as shape, morphology, or biochemical activity and to link these observations over time into biologically meaningful trajectories. Recent advances in optical imaging methods such as light-sheet microscopy now allow researchers to capture volumetric (3D + t) timelapse image data at high-frame rates, with multiple biochemical reporters (Dunsby, 2008; Chen et al., 2014; Kumar et al., 2014; Sapoznik et al., 2020; Yang et al., 2022). As such, we are now in an era where we can generate vast volumes of information-rich experimental imagery more easily than we can extract meaning from the data.

In recent years, machine learning (ML) has had a transformational effect on microscopy data analysis; common image processing tasks such as cell segmentation, image denoising, feature extraction and cell state classification now routinely use a variety of ML-based algorithms (Moen et al., 2019a). ML algorithms can leverage experimental image data to improve robustness and accuracy in the task. However, despite major efforts in developing cell tracking algorithms, extraction of high-fidelity, multi-generational lineages remains a major bottleneck in microscopy image analysis (reviewed in (Wolf et al., 2021)). Most current approaches use the *tracking-by-detection* paradigm, *i.e.*, that the tracking problem is decomposed into two steps i) detection of cells then ii) linking these over time into trajectories.

With the advent of convolutional neural networks (CNNs, (Lecun et al., 1998)), the cell detection step has seen significant progress in recent years. Generalised segmentation and detection algorithms such as U-Net (Ronneberger et al., 2015), Mask R-CNN (He et al., 2017), YOLO (Joseph et al., 2015), Segment Anything (Kirillov et al., 2023) or more specialized cell-specific algorithms such as DeepCell (Van Valen et al., 2016), Cellpose (Stringer et al., 2020) and StarDist (Schmidt et al., 2018) are now able to detect cells with great accuracy, even in complex multidimensional data.

In contrast, many *tracking-by-detection* algorithms have been designed using heuristics based on our prior knowledge of what cells look like, and simple cellular behavior like cell division. As powerful as this approach is, it is often not flexible enough to deal with new or unseen data, and accurate tracking is not the end goal; rather, quantifying the underlying cell biology is. Perhaps a more appealing idea, and the central thesis here, is that advances in ML can be leveraged to learn models of cell behavior by posing cell tracking as the task to be learned.

## 2 Training and validation data

Annotated data are the essential requirement for ML algorithm development, either for training supervised models or evaluating real-world performance. For cell tracking, two types of annotations are required; i) the annotation of individual cells, marking their locations in space and time and ii) annotations describing how these are linked over time. However, acquisition of manual annotations is laborious, time-consuming, with various studies reporting weeks, months or even years of dedicated time spent annotating a medium-sized dataset suitable for model training (Wolff et al., 2018; Caicedo et al., 2019; Ulicna et al., 2021; Malin-Mayor et al., 2022). Ground truth annotations for 3D + t datasets are even more limited as their annotation complexity increases; they are often sparsely annotated or provide a “gold standard” instead. This means that newly-developed tracking approaches are, by definition, benchmarked and validated exclusively against the few tracks included in the gold standard selection, and the model performance is not extensively measured on the entire dataset where it may exhibit some improvements over known tools. For example, the choice of the lineages included in the gold standard could be task-specific, including favouring long, narrow yet complete trees over broken, but richer and wider lineages capturing the diversity of cellular behavior.

In order to increase the quantity and quality of annotated data, there are an increasing number of efforts to crowd-source annotations (Sullivan et al., 2018; Moen et al., 2019b). These efforts have also highlighted the need for active label cleaning for improved dataset quality (Bernhardt et al., 2022). However, with these, and other efforts, the number of high quality datasets is increasing with time, and popular repositories include the Cell Tracking Challenge (CTC), with data from several microscopy modalities (Ulman et al., 2017; Martin et al., 2023)^1^ and the Multiple Object Tracking (MOT) benchmark data capturing diverse cell types from range of model organisms (Anjum and Gurari, 2020)^2^.

## 3 The tracking problem

The tracking data can be represented as a directed acyclic graph (DAG), where the set of cell detections are vertices (*V*, also known as *nodes*). The graph is directed and acyclic due to the arrow of time, and is well suited to representing cell division events. Without any prior knowledge, edges (*E*) are constructed between vertices in successive time points, such that every vertex at time *t* is connected to every vertex at *t* + 1, and so forth. In this case, the full graph of all possible solutions is *G*_hypothesis_ = ⟨*V*, *E*⟩. The goal of a tracking algorithm is to identify a subgraph (*G*_solution_ ⊂ *G*_hypothesis_) that minimises the tracking error and captures the motion and key events, such as mitosis and apoptosis, of every cell in the system. There are two closely related key challenges: (i) detection linking (§3.1) and (ii) lineage reconstruction (§3.2).

### 3.1 Vertices and edges: detection linking

The simplest formulation of the *tracking-by-detection* paradigm uses a greedy assignment strategy. In this case, a cost matrix (**C**) is constructed for all edges between the vertices at time *t* and *t* + 1 (Crocker and Grier, 1996; Jaqaman et al., 2008). Here, **C** yields a simplified version of *G*_hypothesis_; it only considers a successive pair of time points. The goal is to find the optimal set of edges (*G*_solution_) linking vertices, that minimizes the total cost. This is commonly solved as a *Linear Assignment Problem* (LAP), using combinatorial optimization algorithms such as the Hungarian algorithm (also known as the Kuhn–Munkres algorithm (Munkres, 1957)) or the variant Jonker-Volgenant (Jonker and Volgenant, 1987) algorithm. The time complexity of these algorithms is typically *O* (*n* ^3^) making the naïve assignment a costly operation for large numbers of cells.

A central consideration is how to construct the cost matrix **C**. The simplest formulation uses the spatial (*L*_2_, Euclidean) distance between the two vertices. However, this naïve assumption does not capture the heterogeneity of behavior typical in real data, and can produce errors in dense cell populations. More sophisticated cost functions can be formulated, for example, by using the predicted motion of the cell via a Kalman filter (Kalman, 1960; Bise et al., 2011; Bove et al., 2017; Ulicna et al., 2021; Ershov et al., 2022), incorporating local flow (Malin-Mayor et al., 2022) or visual features (He et al., 2015; Bove et al., 2017; Ulicna et al., 2021). However, it seems that there is ample room to incorporate additional features in the construction of **C**. In general, this approach is known as *local* tracking, as although the algorithm is global in space, it is not so in time. In contrast, *global* tracking approaches, consider the full hypothesis graph (all time points) while identifying the optimal set of edges (discussed further in §5.2).

### 3.2 Lineage assembly

**FIGURE 1:**
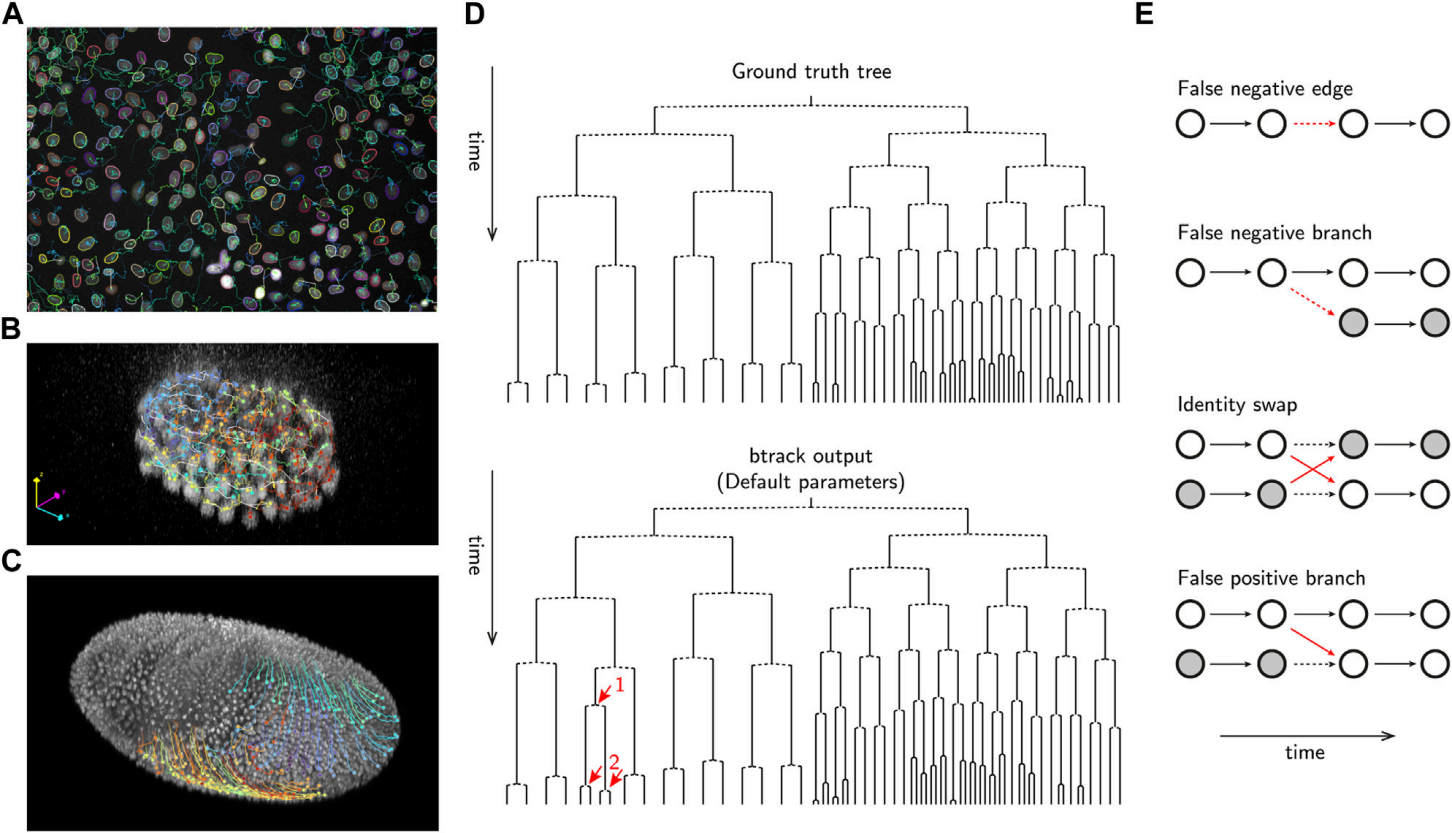
Examples of automated cell tracking and lineaging and evaluation using btrack. **(A)** Tracking MDCK cells in culture (image data from Ulicna et al. (Ulicna et al., 2021)) **(B)** Tracking cells in *C. elegans* early embryo development (image data from Murray et al. (Murray et al., 2006)). **(C)** Tracking cells in *D. melanogaster* embryo development (image data from Amat et al. (Amat et al., 2014)). **(D)** Example btrack (Ulicna et al., 2021) lineage output, using default tracking parameters, on the *C. elegans* dataset from Murray et al. (Murray et al., 2006). The manually annotated ground truth tree is shown for reference. The propagation of a single tracking error, highlighted as a red arrow is shown, demonstrating the complexity of the tracking and lineaging problem. **(E)** Examples of typical errors in automated cell tracking. Vertices are denoted as circles, and correct edges are shown as bold black arrows, errors as red arrows and dashed arrows indicate the ground truth where errors have occurred.

In addition to reconstructing cell *tracks* which follow single-cell trajectories over their lifetime (Figures 1A–C), it is essential to correctly identify cell divisions and the relationships between related cells to precisely reconstruct cell *lineages*. The lineage is a hierarchical organization of single-cell tracks over time, recording the cell division history over up to several generations. Lineages are usually visualized in form of lineage trees (Sandler et al., 2015; E Kuchen et al., 2020), *i.e.*, planar graphical representations from which the ancestral (mother, grandmother, *etc.*) as well as generationally-equal (sister, cousins, *etc.*) relationships can be read (Figure 1D).

Compared to the task of reconstructing single-cell tracks, accurate lineage reconstruction is the most error-prone stage of cell tracking in long-movies. The fidelity of lineage assembly depends on the success of two steps: (i) the formation of full (or partial) trajectories, ideally capturing the cell from division to division, and (ii) the organization of those trajectories into parent-to-children assignments. This process critically depends on the fidelity of object detection, and methods to construct a hypothesis graph that can be evaluated to identify branching events such as mitosis.

## 4 Measuring performance

Measuring tracking errors is critical; metrics are also essential in the design of an effective ML training loop as part of the objective function.

### 4.1 Common tracking errors

Several common errors are observed in automated tracking pipelines (Figure 1E). Fundamentally, any *tracking-by-detection* algorithm is limited by the accuracy of object detection; errors arising from under- and over-segmentation or hallucination, can lead to false negative and false positive cell detections that dramatically impact the construction of *G*_hypothesis_ and therefore the feasibility of potential tracking solutions. For example, missing edges or mitotic detections are common examples of false negatives. A false negative branch occurs when one of the children cells is falsely linked to the parent track, while the other child is initialized as a new track without information about its ancestry. As a result, the tree branches are erroneously prolonged, and the leaf (terminal) cells of the downstream lineage tree path would be determined to reach a lower, and incorrect, generational depth.

On the other hand, it is critical to produce tracks which are not prematurely terminated or truncated, as those could become putative parents (false positive branch), by falsely linking to other tracks arising in their close neighbourhood. In this case, the lineage would incorrectly reach a higher generational depth. Establishing a suite of metrics (See §4.2) is essential for identifying these tracking errors and to enable training new ML models.

### 4.2 Metrics

A few metrics have been proposed for assessing the performance of cell tracking algorithms. Ulman et al. (Ulman et al., 2017) propose a definition of “tracking accuracy” that is based on representations of the ground-truth and predicted cell lineages as DAGs, where accuracy is calculated using a matching measure that assesses the divergence between the ground-truth and predicted graphs (Matula et al., 2015). They also use a “complete tracks” metric that is based on the number of the ground-truth tracks that are correctly tracked, *i.e.*, where a predicted track follows the same cell through all frames of the ground-truth track (Kang et al., 2008).

Further, lineage-specific metrics are required for cell tracking evaluation. For example, Bise et al. (Bise et al., 2011) define mitotic “branching correctness” (MBC) as the proportion of ground-truth cell divisions that are predicted by the tracking model, where a prediction is considered successful if it captures the correct mother-daughter relationship between the cells concerned and, moreover, predicts the timing of the division within a certain tolerance. The MBC and *leaf retrieval score* (LRS, (Ulicna et al., 2021)) are lineage scale metrics. Importantly, in studies where retrieval of the cell relationships is desired, an LRS of 0.75 (*i.e.* 3 out of 4 leaf cells are tracked correctly from start to end of imaging) is more intuitive to the user to benchmark the tracking performance than an often used MOT accuracy metric of 0.97 vs. 0.99. Many of these cell tracking specific metrics are implemented in open-source packages such as Traccuracy^3^.

## 5 Leveraging ML to enhance cell tracking

Naïvely, tracking as few as 10 cells in a movie of 10 frames in length yields a hypothesis graph (*G*_hypothesis_) with a total of 10^9^ possible solutions for each cell. With larger datasets, this naïve approach is computationally infeasible. Luckily, there are constraints on the problem, and not all of these hypotheses are physically possible; cells conform to a set of “rules” defining their behavior, such as movement and division. Rather than “hard-code” these rules into an algorithm, we might approach it as a data-driven problem. Here, ML provides a potential framework to learn these and other cell behaviors. Given a dataset and a set of metrics to enable optimization, tracking can be posed as a learnable task. In addition to detection, a putative model needs two components: i) A way to represent cells from the imaging data and ii) A method of constructing the hypothesis graph from which the tracking solution can be identified.

### 5.1 Learned representations

Many neural network architectures, such as CNNs, operate in a hierarchical fashion, such that high-dimensional information is compressed into a lower-dimensional representation. This makes them a natural tool for the extraction of features representative of the input image data. For cells growing in populations, it is often important to quantitatively describe their immediate neighbourhood (Li et al., 2020; Wang et al., 2020; Fischer et al., 2021; Stirling et al., 2021; Buchner and Valada, 2022). Moreover, single-cell images do not have to be analysed on per-image basis. Indeed, the advantage of time-lapse imaging is that temporal models of cell behavior can encode (or learn) the transitions of states over time (Held et al., 2010; Bove et al., 2017; Soelistyo et al., 2022; Gallusser et al., 2023). Incorporating features such as local (or collective) motion, neighbourhood embeddings or cell state classification can be used to generate rich representations (Bove et al., 2017; Driscoll et al., 2019; Andrews et al., 2021; Gradeci et al., 2021; De Vries et al., 2022; Ko et al., 2022; Malin-Mayor et al., 2022; Yamamoto et al., 2022; Viana et al., 2023). Increasingly, self-supervised methods (such as variational autoencoders) are being used to learn explainable representations directly from the image data ((Zaritsky et al., 2021; Soelistyo et al., 2022; Wu et al., 2022)). As such, the characterization of the temporal landscape of morphological states can help to distinguish heterogeneous cell populations (Freckmann et al., 2022) and diverse cell fates (Soelistyo et al., 2022), or incorporate rules of the tracking problem (Bove et al., 2017). These advances suggest that rich representations of cells can be learned directly from the image data.

### 5.2 Discrete optimization

**FIGURE 2:**
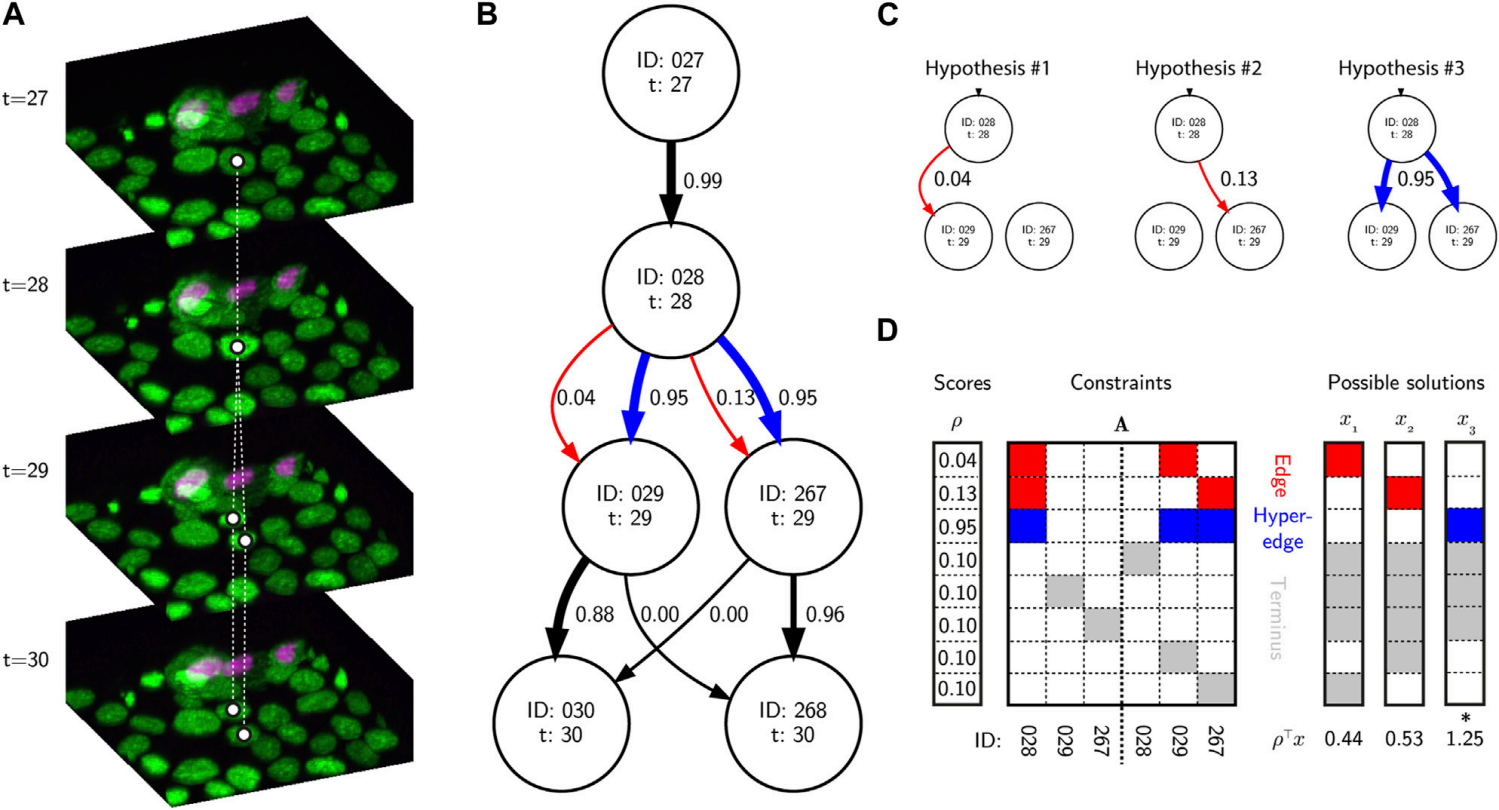
Tracking, graphs and discrete optimization. **(A)** A sequence of image volumes showing a single mitotic branching event highlighted with white circles representing the vertices and dashed lines representing the ground truth edges. **(B)** A simplified directed hypothesis graph of the mitotic event. Each vertex represents a unique cell detection. Edge weights (black and red arrows) and hyperedge weights (blue arrows) are calculated as the posterior probability of linking and branching hypotheses given the evidence, and calculated using btrack. **(C)** There are several hypotheses to account for the appearance of new vertices. In hypotheses 1 & 2, edges link vertex 28 and either 29 or 267 respectively. In hypothesis 3, a hyperedge links vertex 28 to both 29 and 267, representing a mitotic event. **(D)** A simplified ILP optimization problem using the graphical model, and possible solutions. The **A** matrix is *sparse* with non-zero elements colored by hypothesis type (red - edge, blue - hyperedge, grey - terminus). The rows represent individual hypotheses and the columns are the vertex IDs forming part of the hypotheses. The optimal solution (which maximizes *ρ* ^*⊤*^ **x** s.t. $Ax=\vec{1}$ ) is highlighted with an asterisk.

Another major advance in recent years has been the use of discrete optimization methods to identify a *globally* optimal solution graph. One early and very successful approach was to use the Viterbi algorithm (Magnusson et al., 2015), treating cell behavior as a hidden Markov model. Alternatively, optimization can be posed as a *Linear Programming* (LP) problem, *i.e.*, that it is defined by a set of linear inequality constraints that define possible solutions to the problem (Figure 2). The goal is to maximize the value of a linear objective function given these constraints. For example, an Integer LP (ILP) problem can be defined:

$$\begin{array}{cc} \text{maximize} & \rho^{⊤}x\\ \text{subject to} & Ax=b\\ & x\in \left\{0,1\right\} \end{array}$$

Effectively the matrix **A** and vector *ρ* encode a set of hypotheses about potential edges and hyperedges (edges connecting ≥2 vertices, such as a single cell splitting during mitosis) and the likelihood of that hypothesis being correct respectively. Usually this problem is formulated using heuristics, or pre-defined rules, that enable calculation of the likelihood based on the evidence. Since the domain of **x** is binary, the optimization effectively determines the best set of hypotheses that account for all of the observations, while maximizing the total reward. One of the earliest examples was demonstrated by Al-Kofahi et al. (Al-Kofahi et al., 2006), where cell specific hypotheses, such as track splitting were introduced, and this general strategy has been successfully extended with other hypotheses (Bise et al., 2011; Ulicna et al., 2021; Malin-Mayor et al., 2022).

### 5.3 Learning an end-to-end cell tracking model

It should be noted that the construction of *ρ* and **A** are themselves based on *reward functions* and heuristics that are user-provided and therefore parameterized based on our prior knowledge. In btrack we built a *hypothesis engine* that constructs **A** and *ρ* given heuristics and the data; only relevant hypotheses are proposed to limit the size of the ILP problem. The advantage of this approach is that it is computationally cheap and requires little data (acknowledging the general paucity of data; see §2). The disadvantage is that it leads to exponential complexity (ILP is NP-hard) in cases where the problem is ill-posed. In the case of novel or unseen datasets, if the problem is not solvable in a *reasonable* amount of time, it likely means that either the assumptions of the model are incorrect or it is poorly parameterized.

As such, a future goal of an ML system is to reduce the search space by proposing these hypotheses; the aim is not to enumerate all hypotheses, but intelligently suggest a subset that could account for the data. This is analogous to a formal language that describes both the acceptable states of a system but also, potentially the rules that generate those states. Several recent studies have shown that self-supervised methods can identify and predict cellular events from image data alone (Soelistyo et al., 2022; Gallusser et al., 2023). A putative ML-enhanced tracking algorithm could leverage these predictions and associated confidences to construct **A** and *ρ* respectively. Furthermore, recent advances in the fusion of deep-learning and combinatorial solvers provide a route towards achieving this putative end-to-end tracking pipeline (Vlastelica et al., 2019). One can imagine a generalized cell tracking model that has been trained on existing data, which is then further *fine-tuned* for new datasets using transfer learning. Not only does this formulation lead to an implicitly interpretable representation (since **A** is by definition a set of rules), it also enables the exciting prospect of discovering novel cellular dynamics.

## 6 Discussion

Ultimately, the goal of our research is not to create the perfect cell tracking algorithm, rather we want to understand cell behavior in complex biological systems. ML provides a potential framework to learn aspects of cell behavior by posing cell tracking as the learnable task.

Although we have thus-far considered *tracking-by-detection* to be comprised of two separate computational steps, there are efforts in the wider computer vision community to develop end-to-end ML tracking algorithms. In this case, detection and tracking can be posed as a joint learning task, *i.e.*, that the model learns to detect objects and track them simultaneously. This has the advantage of coupling model performance to both steps of the *tracking-by-detection* paradigm. A recent example of this approach are global tracking transformers (Zhou et al., 2022), that couple region proposal networks (Ren et al., 2015) with transformers (Vaswani et al., 2017) to perform semi-global multi-object tracking. These methods however, require very large quantities of training data, and in their current formulation, do not consider important hypotheses such as branching events, making adaption to cell tracking more challenging.

A further requirement in the application of ML for scientific enquiry, is model explainability. Deep neural networks are generally considered “black boxes” whose behavior is difficult to explain in human-understandable terms. Owing to their complexity, these models can typically be explained only with reference to simpler approximation models that mimic the general behavioral features of the more complex model (Guidotti et al., 1802; Rudin et al., 2021). Despite this challenge, the promise of explainable deep learning is considerable, particularly in the domain of scientific inquiry. For example, an explainable end-to-end cell tracking pipeline may allow us to investigate the rules that govern cell movement and behavior. By internalizing an external phenomenon (*e.g.*, cell movement), the model would thereby form a computational representation of that phenomenon that human scientists can investigate. This framework has already been applied with some success to the study of cell fate determination in a cell competition context (Soelistyo et al., 2022).

Finally, the open sharing of trained models, metrics and data, is essential to drive scientific progress. This has been a growing trend in the machine learning community, with the appearance of publicly available repositories such as Scivision^4^, Bioimage. IO (Ouyang et al., 2022) and HuggingFace (Wolf et al., 2020). To maximize ease-of-use, model repositories ensure that each model is accompanied by a standardized description, which includes input and output formats, pre-trained weights and source of training data. Ouyang et al. (Ouyang et al., 2022) note that in order for ML models to be of use to external communities, such as experimental microscopists, models should be accessible via GUI-equipped software, such as Fiji (Johannes et al., 2012) or Napari (Sofroniew et al., 2022). Indeed, effective visualization of tracking data is also an open challenge (examples include Napari, TrackMate (Tinevez et al., 2017; Ershov et al., 2022) and Mastodon^5^).

The promise of ML to help us understand complex biological systems is considerable. Intelligent systems can extract patterns and insights that evade the notice of human scientists, allowing us to investigate domains previously hindered by our limited ability to distil scientific knowledge from large datasets. As such, advances in ML will ultimately enable the automated discovery of novel cellular dynamics.

## Data Availability

The original contributions presented in the study are included in the article/supplementary material, further inquiries can be directed to the corresponding author.

## Footnotes

1
http://celltrackingchallenge.net/datasets/

2
https://motchallenge.net/data/CTMC-v1/

3
https://github.com/Janelia-Trackathon-2023/traccuracy

4
https://sci.vision

5
https://github.com/mastodon-sc/mastodon

## References

[B1] Al-Kofahi O. Radke R. J. Goderie S. K. Temple S. Roysam B. (2006). Automated cell lineage construction: A rapid method to analyze clonal development established with murine neural progenitor cells. Cell. Cycle 5 (3), 327–335. 10.4161/cc.5.3.2426 16434878

[B3] Amat F. Lemon W. Mossing D. P. McDole K. Wan Y. Branson K. (2014). Fast, accurate reconstruction of cell lineages from large-scale fluorescence microscopy data. Nat. Methods 11 (9), 951–958. 10.1038/nmeth.3036 25042785

[B4] Andrews T. G. R. Pönisch W. Paluch E. K. Steventon B. J. Benito-Gutierrez E. Benito-Gutierrez E. (2021). Single-cell morphometrics reveals ancestral principles of notochord development. Development 148 (16), dev199430. 10.1242/dev.199430 34343262 PMC8406538

[B5] Anjum S. Gurari D. (2020). “Ctmc: Cell tracking with mitosis detection dataset challenge,” in 2020 IEEE/CVF Conference on Computer Vision and Pattern Recognition Workshops (CVPRW), Seattle, WA, June 14-19, 2020 (IEEE). 10.1109/cvprw50498.2020.00499

[B7] Bernhardt M. Castro D. C. Tanno R. Schwaighofer A. Tezcan K. C. Monteiro M. (2022). Active label cleaning for improved dataset quality under resource constraints. Nat. Commun. 13 (1), 1161. 10.1038/s41467-022-28818-3 35246539 PMC8897392

[B8] Bise R. Yin Z. Kanade T. (2011). “Reliable cell tracking by global data association,” in 2011 IEEE International Symposium on Biomedical Imaging: From Nano to Macro, Chicago, IL, March 30-April 2, 2011, 1004–1010.

[B6] Bove A. Gradeci D. Fujita Y. Banerjee S. Charras G. AlanLowe R. (2017). Local cellular neighborhood controls proliferation in cell competition. Mol. Biol. Cell. 28 (23), 3215–3228. 10.1091/mbc.e17-06-0368 28931601 PMC5687024

[B9] Buchner M. Valada A. (2022). 3d multi-object tracking using graph neural networks with cross-edge modality attention. Available at: https://arxiv.org/abs/2203.10926 (Accessed March 21, 2022).

[B10] Caicedo J. C. Roth J. Goodman A. Becker T. Karhohs K. W. Broisin M. (2019). Evaluation of deep learning strategies for nucleus segmentation in fluorescence images. Cytom. Part A 95 (9), 952–965. 10.1002/cyto.a.23863 PMC6771982 31313519

[B11] Chen B.-C. Legant W. R. Wang K. Shao L. Milkie D. E. Davidson M. W. (2014). Lattice light-sheet microscopy: Imaging molecules to embryos at high spatiotemporal resolution. Science 346 (6208), 1257998. 10.1126/science.1257998 25342811 PMC4336192

[B13] Crocker J. C. Grier David G. (1996). Methods of digital video microscopy for colloidal studies. J. Colloid Interface Sci. 179 (1), 298–310. 10.1006/jcis.1996.0217

[B14] De Vries M. Dent L. Curry N. Rowe-Brown L. Adam T. Dunsby C. (2022). 3d single-cell shape analysis of cancer cells using geometric deep learning. Available at:https://www.biorxiv.org/content/10.1101/2022.06.17.496550v1 (Accessed June 17, 2022).

[B15] Driscoll M. K. Welf E. S. Jamieson A. R. Dean K. M. Isogai T. Fiolka R. (2019). Robust and automated detection of subcellular morphological motifs in 3d microscopy images. Nat. Methods 16 (10), 1037–1044. 10.1038/s41592-019-0539-z 31501548 PMC7238333

[B16] Dunsby C. (2008). Optically sectioned imaging by oblique plane microscopy. Opt. Express 16 (25), 20306. 10.1364/oe.16.020306 19065169

[B17] E Kuchen E. Becker N. B. Claudino N. Höfer T. (2020). Hidden long-range memories of growth and cycle speed correlate cell cycles in lineage trees. eLife 9, e51002. 10.7554/elife.51002 31971512 PMC7018508

[B18] Ershov D. Phan M. S. Pylvänäinen J. W. Rigaud S. U. Blanc L. L. Orszag A. C. Laine R. F. (2022). TrackMate 7: Integrating state-of-the-art segmentation algorithms into tracking pipelines. Nat. Methods 19 (7), 829–832. 10.1038/s41592-022-01507-1 35654950

[B19] Fischer D. S. Schaar A. C. Theis F. J. (2021). Learning cell communication from spatial graphs of cells. Available at: https://www.biorxiv.org/content/10.1101/2021.07.11.451750v1.full (Accessed July 12, 2021). 10.1038/s41587-022-01467-z PMC10017508 36302986

[B20] Freckmann E. C. Sandilands E. Cumming E. Neilson M. Román-Fernández A. Nikolatou K. (2022). Traject3d allows label-free identification of distinct co-occurring phenotypes within 3d culture by live imaging. Nat. Commun. 13 (1), 5317. 10.1038/s41467-022-32958-x 36085324 PMC9463449

[B21] Gallusser B. Stieber M. Weigert M. (2023). Self-supervised dense representation learning for live-cell microscopy with time arrow prediction. Available at: https://arxiv.org/abs/2305.05511 (Accessed May 9, 2023).

[B22] Gradeci D. Anna B. Vallardi G. Lowe A. R. Banerjee S. Charras G. (2021). Cell-scale biophysical determinants of cell competition in epithelia. eLife 10, e61011. 10.7554/elife.61011 34014166 PMC8137148

[B23] Guidotti R. Anna M. Ruggieri S. Franco T. Pedreschi D. Giannotti F. A survey of methods for explaining black box models. Available at; http://arxiv.org/abs/1802.01933 (Accessed February 6, 2018).

[B24] He K. Gkioxari G. Dollar P. Girshick R. (2017). “Mask R-CNN,” in 2017 IEEE International Conference on Computer Vision (ICCV), Venice, Italy, October 22-29, 2017 (IEEE).

[B25] He K. Zhang X. Ren S. Sun J. (2015). Deep residual learning for image recognition. Available at: http://arxiv.org/abs/1512.03385 (Accessed December 10, 2015).

[B26] Held M. Schmitz M. H. A. Fischer B. Walter T. Neumann B. Olma M. H. (2010). Cellcognition: Time-resolved phenotype annotation in high-throughput live cell imaging. Nat. methods 7 (9), 747–754. 10.1038/nmeth.1486 20693996

[B27] Jaqaman K. Loerke D. Mettlen M. Kuwata H. Grinstein S. Schmid S. L. (2008). Robust single-particle tracking in live-cell time-lapse sequences. Nat. Methods 5 (8), 695–702. 10.1038/nmeth.1237 18641657 PMC2747604

[B28] Johannes S. Arganda-Carreras I. Frise E. Kaynig V. Longair M. Tobias P. (2012). Fiji: An open-source platform for biological-image analysis. Nat. Methods 9 (7), 676–682. 10.1038/nmeth.2019 22743772 PMC3855844

[B29] Jonker R. Volgenant A. (1987). A shortest augmenting path algorithm for dense and sparse linear assignment problems. Computing 38 (4), 325–340. 10.1007/bf02278710

[B30] Joseph R. Divvala S. Girshick R. Ali F. (2015). You only look once: Unified, real-time object detection. Available at:https://arxiv.org/abs/1506.02640 (Accessed June 8, 2015).

[B31] Kalman R. E. (1960). A new approach to linear filtering and prediction problems. J. Basic Eng. 82 (1), 35–45. 10.1115/1.3662552

[B32] Kang L. Chen M. Kanade T. Miller E. D. Weiss L. E. Campbell P. G. (2008). Cell population tracking and lineage construction with spatiotemporal context. Med. image Anal. 12 (5), 546–566. 10.1016/j.media.2008.06.001 18656418 PMC2670445

[B33] Ko S. Çevrim Ç. Averof M. (2022). Tracking cell lineages in 3d by incremental deep learning. eLife 11, 69380. 10.7554/elife.69380 PMC8741210 34989675

[B2] Kirillov A. Mintun E. Ravi N. Mao H. Rolland C. Gustafson L. (2023). Segment anything. Available at: https://arxiv.org/abs/2304.02643 (Accessed April 5, 2023).

[B34] Kumar A. Wu Y. Christensen R. Chandris P. Gandler W. McCreedy E. (2014). Dual-view plane illumination microscopy for rapid and spatially isotropic imaging. Nat. Protoc. 9 (11), 2555–2573. 10.1038/nprot.2014.172 25299154 PMC4386612

[B35] Lecun Y. Bottou L. Bengio Y. Haffner P. (1998). Gradient-based learning applied to document recognition. Proc. IEEE 86 (11), 2278–2324. 10.1109/5.726791

[B36] Li J. Gao X. Jiang T. (2020). “Graph networks for multiple object tracking,” in 2020 IEEE Winter Conference on Applications of Computer Vision (WACV), Snowmass, CO, March 1-5, 2020, 708–717.

[B37] Magnusson K. E. G. Jalden J. Gilbert P. M. Blau H. M. (2015). Global linking of cell tracks using the viterbi algorithm. IEEE Trans. Med. Imaging 34 (4), 911–929. 10.1109/tmi.2014.2370951 25415983 PMC4765504

[B38] Malin-Mayor C. Hirsch P. Guignard L. McDole K. Wan Y. Lemon W. C. (2022). Automated reconstruction of whole-embryo cell lineages by learning from sparse annotations. Nat. Biotechnol. 41 (1), 44–49. 10.1038/s41587-022-01427-7 36065022 PMC7614077

[B39] Martin M. Ulman V. Delgado-Rodriguez P. Gómez de Mariscal E. Nečasová T. Fidel A. (2023). The cell tracking challenge: 10 years of objective benchmarking. Nat. Methods. 10.1038/s41592-023-01879-y PMC10333123 37202537

[B40] Matula P. Martin M. Sorokin D. V. Matula P. Ortiz de Solórzano C. Kozubek M. (2015). Cell tracking accuracy measurement based on comparison of acyclic oriented graphs. PLOS ONE 10 (12), e0144959. 10.1371/journal.pone.0144959 26683608 PMC4686175

[B41] Moen E. Bannon D. Kudo T. Graf W. Covert M. Van Valen D. (2019a). Deep learning for cellular image analysis. Nat. Methods 16 (12), 1233–1246. 10.1038/s41592-019-0403-1 31133758 PMC8759575

[B42] Moen E. Borba E. Miller G. Schwartz M. Bannon D. Koe N. (2019b). Accurate cell tracking and lineage construction in live-cell imaging experiments with deep learning. Available at; https://www.biorxiv.org/content/10.1101/803205v2 (Accessed October 14, 2019).

[B43] Munkres J. (1957). Algorithms for the assignment and transportation problems. J. Soc. Industrial Appl. Math. 5 (1), 32–38. 10.1137/0105003

[B44] Murray J. I. Bao Z. J Boyle T. Waterston R. H. (2006). The lineaging of fluorescently-labeled caenorhabditis elegans embryos with StarryNite and AceTree. Nat. Protoc. 1 (3), 1468–1476. 10.1038/nprot.2006.222 17406437

[B45] Ouyang W. Beuttenmueller F. Gómez-de Mariscal E. Pape C. Burke T. Garcia-López-de Haro C. (2022). BioImage model zoo: A community-driven resource for accessible deep learning in BioImage analysis. Available at: https://www.biorxiv.org/content/10.1101/2022.06.07.495102v1 (Accessed June 8, 2022).

[B46] Ren S. He K. Girshick R. Sun J. (2015). Faster r-cnn: Towards real-time object detection with region proposal networks. Available at: https://arxiv.org/abs/1506.01497 (Accessed June 4, 2015). 10.1109/TPAMI.2016.2577031 27295650

[B47] Ronneberger O. Fischer P. Brox T. (2015). U-net: Convolutional networks for biomedical image segmentation. Available at: http://arxiv.org/abs/1505.04597 (Accessed June 4, 2015).

[B48] Rudin C. Chen C. Chen Z. Huang H. Semenova L. Zhong C. (2021) Interpretable machine learning: Fundamental principles and 10 grand challenges. Available at: http://arxiv.org/abs/2103.11251 (Accessed June 4, 2021).

[B49] Sandler O. Mizrahi S. P. Weiss N. Agam O. Simon I. Nathalie Q. (2015). Lineage correlations of single cell division time as a probe of cell-cycle dynamics. Nature 519 (7544), 468–471. 10.1038/nature14318 25762143

[B50] Sapoznik E. Chang B. J. Huh J. Ju R. J. Azarova E. V. Pohlkamp T. (2020). A versatile oblique plane microscope for large-scale and high-resolution imaging of subcellular dynamics. eLife 9, e57681. 10.7554/elife.57681 33179596 PMC7707824

[B51] Schmidt U. Weigert M. Broaddus C. Myers G. (2018). “Cell detection with star-convex polygons,” in Medical image computing and computer assisted intervention – miccai 2018 (Cham: Springer International Publishing), 265–273.

[B12] Soelistyo C. J. Vallardi G. Charras G. Lowe A.R. (2022). Learning biophysical determinants of cell fate with deep neural networks. Nat. Mach. Intell. 4 (7), 636–644. 10.1038/s42256-022-00503-6

[B52] Sofroniew N. Lambert T. Evans K. Nunez-Iglesias J. Bokota G. Winston P. (2022). napari: a multi-dimensional image viewer for python. Available at: https://zenodo.org/record/7276432 (Accessed November 3, 2022).

[B53] Stirling D. R. Swain-Bowden M. J. Lucas A. M. Carpenter A. E. Cimini B. A. Allen G. (2021). CellProfiler 4: Improvements in speed, utility and usability. BMC Bioinforma. 22 (1), 433. 10.1186/s12859-021-04344-9 PMC8431850 34507520

[B54] Stringer C. Wang T. Michaelos M. Pachitariu M. (2020). Cellpose: A generalist algorithm for cellular segmentation. Nat. Methods 18 (1), 100–106. 10.1038/s41592-020-01018-x 33318659

[B55] Sullivan D. P. Winsnes C. F. Åkesson L. Martin H. Wiking M. Schutten R. (2018). Deep learning is combined with massive-scale citizen science to improve large-scale image classification. Nat. Biotechnol. 36 (9), 820–828. 10.1038/nbt.4225 30125267

[B56] Tinevez J.-Y. Perry N. Johannes S. GenevieveHoopes M. Laplantine E. Bednarek S. Y. (2017). TrackMate: An open and extensible platform for single-particle tracking. Methods 115, 80–90. 10.1016/j.ymeth.2016.09.016 27713081

[B57] Ulicna K. Vallardi G. Charras G. Lowe A. R. (2021). Automated deep lineage tree analysis using a Bayesian single cell tracking approach. Front. Comput. Sci. 3, 734559. 10.3389/fcomp.2021.734559

[B58] Ulman V. Martin M. Magnusson K. E. G. Ronneberger O. Haubold C. Harder N. (2017). An objective comparison of cell-tracking algorithms. Nat. Methods 14 (12), 1141–1152. 10.1038/nmeth.4473 29083403 PMC5777536

[B59] Van Valen D. A. Kudo T. Lane K. M. Macklin D. N. Quach N. T. DeFelice M. M. (2016). Deep learning automates the quantitative analysis of individual cells in live-cell imaging experiments. PLOS Comput. Biol. 12 (11), e1005177. 10.1371/journal.pcbi.1005177 27814364 PMC5096676

[B60] Vaswani A. Shazeer N. Parmar N. Uszkoreit J. Jones L. Gomez A. N. (2017). “Attention is all you need,” in Advances in neural information processing systems. Editors Guyon I. Von Luxburg U. Bengio S. Wallach H. Fergus R. Vishwanathan S. (New York, NY: Curran Associates, Inc.).

[B61] Viana M. P. Chen J. Knijnenburg T. A. Vasan R. Yan C. Arakaki J. E. (2023). Integrated intracellular organization and its variations in human iPS cells. Nature 613 (7943), 345–354. 10.1038/s41586-022-05563-7 36599983 PMC9834050

[B62] Vlastelica M. Paulus A. Musil V. Martius G. Rolínek M. (2019). Differentiation of blackbox combinatorial solvers. Available at:https://arxiv.org/abs/1912.02175 (Accessed December 4, 2019).

[B63] Wolf S. Wan Y. McDole K. (2021). Current approaches to fate mapping and lineage tracing using image data. Development 148 (18), dev198994. 10.1242/dev.198994 34498046

[B64] Wolf T. Debut L. Sanh V. Chaumond J. Clement D. Anthony M. (2020). HuggingFace’s transformers: State-of-the-art natural language processing. Available at: https://arxiv.org/abs/1910.03771 (Accessed October 9, 2019).

[B65] Wolff C. Tinevez J. Y. Tobias P. Stamataki E. Harich B. Guignard L. (2018). Multi-view light-sheet imaging and tracking with the mamut software reveals the cell lineage of a direct developing arthropod limb. eLife 7, e34410. 10.7554/eLife.34410 29595475 PMC5929908

[B66] Wu Z. Chhun B. B. Popova G. Guo S.-M. Kim C. N. Yeh L.-H. (2022). DynaMorph: Self-supervised learning of morphodynamic states of live cells. Mol. Biol. Cell. 33 (6), 1939–4586. 10.1091/mbc.E21-11-0561 PMC9265147 35138913

[B67] Yamamoto T. Cockburn K. Greco V. Kawaguchi K. (2022). Probing the rules of cell coordination in live tissues by interpretable machine learning based on graph neural networks. PLOS Comput. Biol. 18 (9), e1010477. 10.1371/journal.pcbi.1010477 36067226 PMC9481156

[B68] Yang B. Lange M. Millett-Sikking A. Zhao X. Bragantini J. VijayKumar S. (2022). DaXi—High-resolution, large imaging volume and multi-view single-objective light-sheet microscopy. Nat. Methods 19 (4), 461–469. 10.1038/s41592-022-01417-2 35314838 PMC9007742

[B69] Wang Y. Kitani K. Weng X. (2020). Joint object detection and multi-object tracking with graph neural networks. Available at: https://arxiv.org/abs/2006.13164 (Accessed June 23, 2020).

[B70] Zaritsky A. Jamieson A. R. Welf E. S. Andres N. Cillay J. Eskiocak U. (2021). Interpretable deep learning uncovers cellular properties in label-free live cell images that are predictive of highly metastatic melanoma. Cell. Syst. 12 (7), 733–747.e6. 10.1016/j.cels.2021.05.003 34077708 PMC8353662

[B71] Zhou X. Yin T. Koltun V. Krähenbühl P. (2022). Global tracking transformers. Available at: https://arxiv.org/abs/2203.13250 (Accessed March 24, 2022).

